# Rethinking marine plastics pollution: Science diplomacy and multi-level governance

**DOI:** 10.1177/00208523231183909

**Published:** 2023-07-04

**Authors:** Macarena Beltran, Benny Tjahjono, Thomas Noto Suoneto, Rakyan Tanjung, Jorge Julião

**Affiliations:** 2706 Coventry University, UK; 2706 Coventry University, UK; Indonesian Chamber of Commerce and Industry (KADIN), Indonesia; 2706 Coventry University, UK; 59207 Universidade Católica Portuguesa, Portugal

**Keywords:** Science diplomacy, multi-level governance, environmental governance, international relations, marine pollution, plastics pollution

## Abstract

Although science diplomacy has been gaining relevance in foreign policy to solve environmental challenges, critical questions concerning what different instruments mean under the term ‘science diplomacy’ and whether science diplomacy does contribute to the progress in solving environmental issues remain unanswered. We explore those questions by linking science diplomacy salient features through documentary analysis of international instruments addressing the plastics pollution in the ocean. We find that from a science diplomacy and multi-level governance perspective, the responsibilities and capabilities of emergent actors of cross-level governance are also important, leading to more attention being paid to changes in the role of national authorities, away from passive leadership to cross-cutting coalitions supported by the salient features of science diplomacy, and redefining dominant discursive approaches that have framed plastics waste.

**Points for practitioners:**

As a result of the cost and limitations of the current international mechanisms, there is currently no incentive for individual countries to take action against marine plastic pollution.

Science diplomacy and multi-level governance can contribute to international cooperation, foreign policy and national strategies.

Leading efforts to engage countries with fewer scientific and technological capabilities could benefit countries’ foreign policy.

## Introduction

Science diplomacy (SD) has gained a foothold in foreign policy to solve global environmental issues. Indeed, SD has been envisioned as one of the policies for the European Commission to collectively address huge challenges such as environmental degradation (Moedas, 2016). The case study of substances depleting the ozone layer has been exemplified as a successful case that interlinks science and diplomacy. Science was used as a source of early warning for the depletion of the ozone layer, a force for change, a confidence-building activity, a measure of regulatory performance and a tool for diplomacy, specifically under the 1987 Montreal Protocol (Sarma and Andersen, 2011). However, the complex problem caused by plastics pollution in the ocean –impacting livelihoods, health, climate change, socio-economy and geopolitics – is one of the main unresolved global environmental issues. The lessons learned in the Montreal Protocol could be transferred to solve plastics pollution. Yet, so far, it has not been sufficiently clarified what different international instruments mean under the term *science diplomacy* in solving the issue of plastics pollution in the ocean.

The existing international, regional and national initiatives and policies have not produced the expected results (Agamuthu et al., 2019). There are multiple regulatory gaps and problems in international ocean plastics governance (Chen, 2015; Gold et al., 2014), including the lack of harmonisation of international laws, weak coherence across national policies, weak coordination of international organisations and inadequate science–policy interaction (Ferraro and Failler, 2020). Analyses of governance emphasise the importance of concurrent instruments at the different levels of governance (Chen, 2015; Gold et al., 2014), supra-national, international and national, to combat plastics pollution in the ocean. However, this poses significant challenges for policymakers, meaning the long-term response, the potential incoherence within and between policy goals and policy means, and the governance ability (Rayner and Howlett, 2009).

Therefore, drawing from SD, we aim to enhance the understanding of the role of international instruments in reducing and mitigating plastic marine pollution. In particular, this paper seeks to address what the different international instruments for marine plastics pollution mean under the term *science diplomacy* and whether SD actually contributes to progress in solving the issue. We contend that SD can shed light on the ocean's plastics pollution problem by paying more attention to cross-responsibilities and leadership capabilities across the tiers of governance (multi-level governance (MLG) Type II) and redefining dominant discursive approaches that have framed plastics waste.

The remainder of the paper is structured as follows. The second section summarises previous literature on SD as international instruments for marine governance and presents the analytical framework conceptualising the link between SD salient features and marine plastics pollution. The third section provides the research approach to guide the analysis of the research. The fourth section presents the findings and discusses the opportunities of current global plastics pollution governance and the complex solution-making process within the plastics pollution-related agenda items from an SD perspective. This is then followed by the description of the research implications, limitations, and suggestions for the future research, before the conclusion of the research in the fifth section.

## International institutions and marine plastics pollution

### Literature review on SD on international instruments on marine governance

The urgency and relevance of global challenges have triggered an increased demand for international cooperation in science, technology and innovation (Boekholt et al., 2009). The continuing global challenges and the role of science in solving them have continued to demand the integration of international science cooperation (Turekian, 2018). Science as a broad basis for diplomacy has significantly increased in relevance to dealing with environmental problems.

Broadly speaking, literature on SD linked to marine environmental issues can be grouped into articles dealing with marine biodiversity, conservation and protected areas (Harden-Davies, 2018; Kleinhaus et al., 2020; Teff-Seker et al., 2020), management of the polar regions, Atlantic ocean (Pan and Huntington, 2016; Polejack et al., 2021) and international instruments (Paglia, 2021; Polejack, 2021). In the literature review, only a few articles were found in which marine-related international instruments had been explicitly mentioned in connection to SD.

Polejack (2021) discussed the importance of SD to reconcile different positions driven by the gap between scientific capacities and access to research infrastructure between developed and developing countries at the negotiations in the United Nations Convention on the Law of the Sea (UNCLOS). For instance, states were organised into clusters (e.g. the G77 plus China, the landlocked states, etc.), and trade-offs among states led to the agreement of compromises in the Convention. Yet, the author noted, research capacity and technology transfer have yet to reach a desirable level. In order to overcome historical obstacles (such as the scientific capacity gap) and progress long-awaited initiatives, such as mapping and forecasting the ocean, SD will be pivotal. Paglia (2021) acknowledges that environmental diplomacy was closely allied with science at the 1972 United Nations Conference on the Human Environment (UNCHE). In terms of science in diplomacy, a Swedish scientist-diplomat facilitated the development of a scientific understanding of widespread environmental degradation's societal threat to inform foreign policy, while diplomacy for science emphasised the importance of mobilising knowledge and experience for developing countries through appropriate international cooperation.

### Analytical framework: conceptualising the link between SD salient features and marine plastics pollution

SD has its roots in the realms of security (Turekian, 2018). After the conflict between the United States and Iraq and the 9/11 tragic events (Lord and Turekian, 2007), the American Association for the Advancement of Science (AAAS) increased its focus on SD to improve its weakened foreign policy and diplomacy engagement with the Middle East and North Africa region (Turekian, 2018). In 2010 the AASS, along with the British Royal Society, defined SD as a concept in three ways: (a) science in diplomacy; (b) diplomacy for science; (c) science for diplomacy (Royal Society, 2010) (Table 1). Applying these three ways to conceptualise SD is grounded on the understanding that science and politics are the dominant elements. However, SD should be distinguished from its scientific and political context.

**Table 1.** Science diplomacy definitions by the Royal Society (2010).

Science diplomacy concepts	Description
Science in diplomacy	Scientific advice is used to inform foreign policy.
Diplomacy for science	Political capital is used to advance scientific research.
Science for diplomacy	Scientific cooperation is used to improve international relations.

The definitions by the Royal Society have been evolving to include different features and views of SD. For instance, Fähnrich (2017) pointed out that at first glance, science for diplomacy and diplomacy for science may be seen as two sides of the same coin; however, when he examined the views of scholars from a German SD programme, not all of them are willing to follow the government views. Besides, other authors call for an extended dimension of science for diplomacy that seeks to reflect the political influence sought and exerted under the term SD in which countries in the international arena represent their interests with a base of knowledge acquired by the scientific method (Gluckman et al., 2017; Ruffini, 2020; Turekian, 2018).

Overall, all these SD dimensions represent ways in which states, or relevant actors, use scientific knowledge to help build international partnerships to address common problems (Patman and Davis, 2017), driven by a subset of public diplomacy (Copeland, 2015). However, the interaction between science, technology and international affairs is embedded in a complex combination of political, legal and cultural forces (Weiss, 2015). Our methodology was first set to apply the different mainstream definitions of SD to marine plastics governance documents. However, we examined the main salient features of SD dimensions to establish a common understanding, while acknowledging the evolving definitions of SD. SD is a broad and evolving concept representing a wide range of meanings insufficiently captured by the Royal Society dimensions. This paper does not intend to produce a new definition; rather, it intends to address what different instruments mean under the term SD.

**Figure 1.**
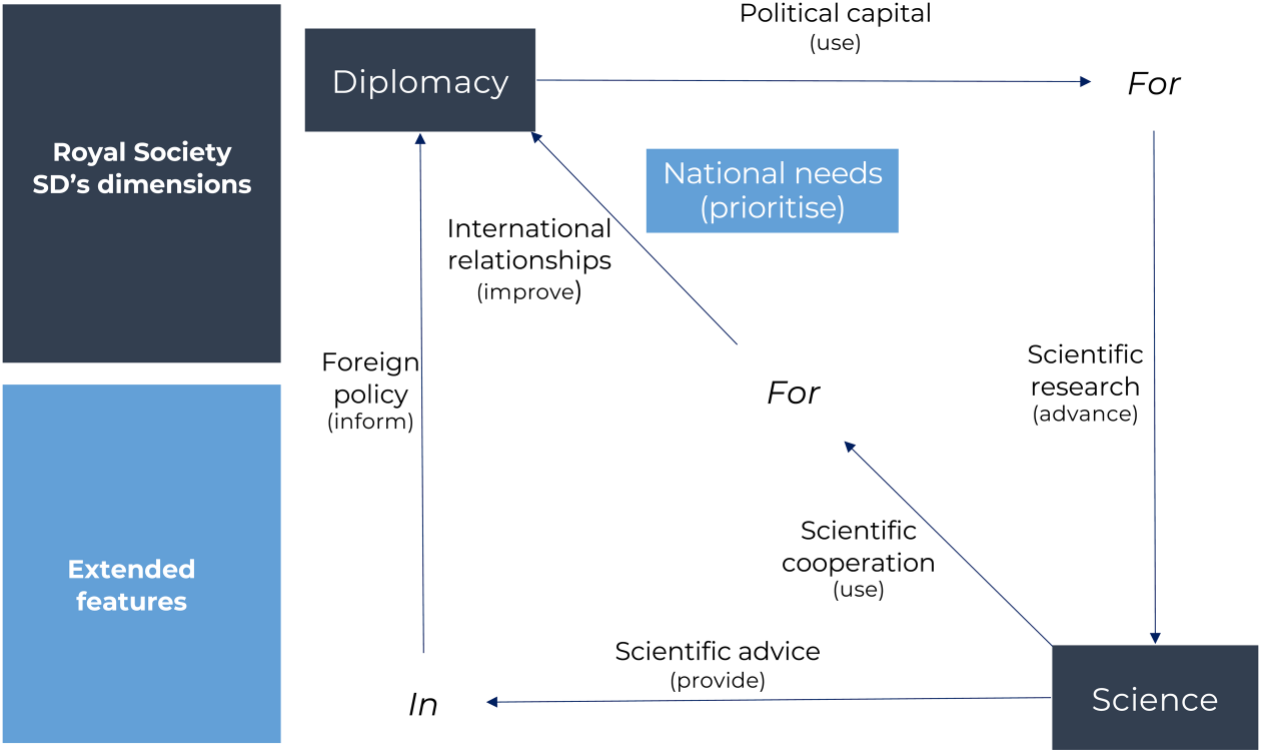
Main salient features of Royal Society dimensions and extended features.

At this point, we added extended dimensions’ features that emerged from definitions that recall the political influence sought and exerted under the term *science diplomacy*, in which countries in the international arena represent their own interests with a base of knowledge acquired by the scientific method (Gluckman et al., 2017; Ruffini, 2020; Turekian, 2018). We labelled the new salient feature under the science for diplomacy dimension (see Figure 1). As a starting point, the framework for analysis distinguishes the salient features of the Royal Society definitions for science for diplomacy, science in diplomacy and diplomacy for science. The features that describe the functions of *scientists* include: ‘scientific cooperation’, providing ‘scientific advice’ and advances in ‘scientific research’, while the features that describe the functions of *diplomats* can be summarised as improvement of ‘international relationships’, informing ‘foreign policy’ and use of ‘political capital’. However, those features fail to capture the use of SD to represent a country's interest using scientific knowledge (see for example descriptions from Gluckman et al., 2017; Ruffini, 2020; Turekian, 2018). In order to represent this salient feature, the dimension of SD was chosen as an umbrella. The new feature was labelled as prioritising ‘national needs’ (see Figure 1).

The following list includes the main salient features from the dimensions described in the second section that can be used either individually or collectively to characterise the functions of SD:

- Science:
  - Scientific cooperation
  - Provide scientific advice
  - Advance of scientific research.
- Diplomacy:
  - Improvement of international relationships
  - Inform foreign policy
  - Use of political capital
  - Prioritisation of a country's national needs.

This typology of SD dimensions somewhat concretises the salient features by listing them under the concepts of science and diplomacy, and it is used to recognise the type of collaboration of different actors, such as the main international organisations, often crucial to achieving environmental agreements, protocols, regulations recognising the establishment of the international and regional instruments would not have been possible without pre-existing diplomatic links and agreements.

## Research approach

To answer the research questions, this study tested the analytical framework in the context of international instruments related to managing marine plastic pollution issues driven by organisations that operate at the supra-level, such as the International Maritime Organization (IMO) in its role as facilitator for prevention and control of marine pollution from ships (IMO, 2019a) and the United Nations (UN) agency in environmental affairs – the United Nations Environment Programme (UNEP) (UNEP, 2023).

A documentary research review and analysis (Ahmed, 2010; Tight, 2019) was employed to explore the role of the international institutions in tackling marine plastic pollution. This methodological approach is recommended when data may not be available in other forms, organisations are required to publish normative documents, documents are reliable data sources for comparative policy, ethical issues are not an immediate issue (e.g. privacy issues), documents are prepared before the research, and thus are ‘non-reactive’, documents may provide supplementary data that can be used to contextualise or clarify other methods of data collection, and documentary analysis may also help to inform different stages of the research process (Shaw et al., 2004). Although documents are not necessarily a neutral source and may represent a partial account of the problem (Atkinson and Coffey, 2010), and the analysis ‘*may also tend to be positivist in philosophy, taking at face value the “apparent” meanings of texts*’ (Shaw et al., 2004: 259). Yet this methodology still has value to represent the statements of policies and strategies at particular points in time through an interpretative analysis.

The material reviewed in detail comprised seven publications of instruments and five webpages of organisations at the supra-level driven by, published or working in collaboration with the IMO and UN (see Annex 1–2). Firstly, due to their important role in managing marine pollution issues, the IMO documentary online catalogue (http://imo.iii.com/) was used to retrieve the documents by using the keywords ((“convention” or “protocol”) AND (“plastic” or “waste”) AND (“pollution”)) in English from 2000 onwards under the category of any field (see Annex 3). A list of 38 documents was retrieved; 28 documents were available online. After reading the summary/abstract, 16 were not included because documents were duplicated (2), books/yearbooks and other guides (12) or regional agreements (2).

Secondly, a refinement of the selection of publications (12) was accomplished, considering the following eligibility criteria on – the document is important to understand the role of international instruments in addressing plastic marine pollution. Documents were selected and grouped according to their relation to the main marine legislation (nine). The latest versions of the documents and related treaties were sought (i.e. London Convention (LC)/London Protocol (LP) (two), International Convention for the Prevention of Pollution from Ships (MARPOL) (two), UNCLOS (two), Basel Convention (one)). Thirdly, these documents (seven) were revised along the main international organisation web pages (e.g. IMO.org, Basel.int, etc.) (see Annex 2). The interpretation and summary of the potential impact on combating marine plastics pollution of these instruments was made in Excel by two researchers independently (see Annex 3 and Table 2):

- Milestone and rationale
- Sources of plastics pollution covered in the regulation
- Plastics materials included under the regulation
- Compliance.

**Table 2.** Summary of revised conventions and protocols related to ocean plastics pollution (authors’ elaboration).

International instruments	Impact				
	Milestone	Rationales	Sources of plastics pollution covered in the regulation	Plastics materials included	Compliance
Convention on the Prevention of Marine Pollution by Dumping of Wastes and Other Matter (London Convention) (International Marine Organization (IMO), 2016)	Adopted in 1972, entered into force in 1975.	Ensure materials dumped into the ocean, including plastics, do not contain harmful materials.	Limited plastic waste discarded from ships (direct disposal) – covered under dumping of waste and other materials.	‘Persistent plastics and other persistent synthetic materials, for example, netting and ropes’ (Annex I(4)).	State parties are responsible for implementing international rules within their own jurisdiction.
International Convention for the Prevention of Pollution from Ships 1973, modified with the Protocol of 1978 (MARPOL) (IMO, 2022a)	1973 (Convention); 1978 (Protocol) entered into force in 1983.	Safeguard the marine environment from harmful substances, oil pollution, garbage, sewage, air pollution and emission from ships.	Limited plastic garbage discarded from ships – covered under prevention of *pollution by garbage* from ships.	‘solid material which contains as an essential ingredient one or more high molecular mass polymers and which is formed (shaped) during either manufacture of the polymer or the fabrication into a finished product by heat and/or pressure’ (Annex 5, Regulation 1(13)).	State parties are responsible for implementing the current Convention and its Annexes. Preventing pollution should be the focus (IMO, 2013). Tracking waste management is difficult due to the varying amounts of garbage from one ship type to another (IMO, 2013).
The United Nations Convention on the Law of the Sea (UNCLOS) (UN, 2001; UNCLOS, 1982).	Adopted in 1982, entered into force 1994.	Define rights, obligations, boundaries of exploration and exploitation for the ocean environment.	A major focus is preventing, mitigating, and controlling marine pollution.	Includes pollution from land-based sources (Article 207), pollution by dumping (Article 210) and vessels (Article 211) and others, *but no specifics regarding plastics.*	Each state shall take, individually or jointly, all measures necessary to prevent, reduce, and control pollution of the marine environment (Article 194(1)). The current state of dumping at sea by states is difficult to measure (UN, 2001).
Basel Convention (United Nations Environment Programme, 2014)	Adopted in 1989, entered into force in 1992.	Control the disposal of hazardous waste generation, restrict and regulate the transboundary movement of hazardous waste shipments.	Hazardous plastic waste subject to transboundary movement (Basel Convention, 2019a), excluding plastic waste from normal operation of ships.	In 2019, plastics were included as hazardous waste (Decision BC-14/12). Differentiation of hazardous/non-hazardous waste is based on: whether the plastic waste shipment is ‘almost free of contamination’, and is ‘almost exclusively’ composed of one polymer (Basel Convention, 2019b).	All parties are: - Required to ban imports and exports of hazardous waste; - Forbidden from trading with non-parties; - Responsible for reducing their waste generation (Article 4). Legal ambiguity to define hazardous or non-hazardous plastic waste – international and national specifications provide a reference for definitions.
London Protocol (IMO, 2016)	Adopted in 1996, entered into force in 2006.	Tighten the London Convention and build more restrictive policies for its signatories and parties.	Limited plastics discarded from ships – covered under dumping of waste and other materials.	All dumping is prohibited unless it is explicitly allowed, including plastics. It does not apply to land-based sources.	Contracting Parties shall apply a ‘precautionary approach to environmental protection from dumping of wastes’ (Article 3(1)).

Fourthly, SD features (Figure 1) contained in each instrument were analysed, interpreted and represented in Table 3 (see Annex 3).

**Table 3.** Features of science diplomacy dimensions applied to international instruments at supra-national levels (authors’ elaboration).

MLG Instruments	Science			Diplomacy			
	Scientific cooperation (use)	Scientific advice (provide)	Scientific research (advance)	International relationships (improve)	Foreign policy (inform)	Political capital (use)	Prioritise national needs
London Convention (IMO, 2016)	Promotes collaboration between the organisation and other international organisations in the scientific research, training and equipment (Articles VIII, IX).	Receives scientific advice from different organisations (rather than provide) including consultative or special meetings of the contracting parties and Group of Experts on the Scientific Aspects of Marine Environmental Protection (GESAMP).	By promoting and using scientific research, science is indirectly advanced.	Provides a platform for collaboration with the international community to control marine pollution sources. Promotes potential interregional agreements.	Foreign policy of state parties is influenced by this convention.	Preparatory diplomacy to achieve agreements and amendments.	87 states are parties to this convention (56.67% World Tonnage) (IMO, 2022b).
MARPOL (IMO, 2022a)	Promotes technical and research cooperation, including training of technical and scientific personnel, monitoring and control equipment, reception facilities, prevention and control measures, and research promotion.	Receives scientific/technical advice (rather than provide) from MARPOL parties, IMO-related organisations (e.g. GESAMP), etc.		It has mandatory and optional protocols that could lead to greater cooperation. Marine Environment Protection Committee (MEPC) provides a platform to address environmental issues.			155 countries have signed up Annex V (Prevention of pollution from ships by garbage) (98.60% World Tonnage) (IMO, 2022b).
UNCLOS (UN, 2001)	Promotes scientific research and technical collaboration to developing states for the protection and preservation of the marine environment and the prevention, reduction and control of marine pollution (Article 202).	Promotes the acquisition of scientific advice, emphasises cooperation (Article 201), and promotes marine scientific research (Article 239) by states directly or through international organisations (e.g. SOCA, GESAMP, ICSPRO).		Presents opportunities for international collaboration on a global or regional basis, directly or through international organisations.			168 parties, 157 signatories (UNTC, 2023)
Basel Convention (UNEP, 2014)	Promotes scientific cooperation to improve and achieve management of hazardous and other wastes (e.g., making available information, technology and management systems) (Article 10(1)).	Receives and transmits information to and from parties regarding technical assistance, training, technical and scientific know-how, advice and expertise, and availability of resources (Article 16). Amendments are based on scientific advice (Article 17).		Promotes bilateral, multilateral, or regional agreements or arrangements regarding transboundary waste (Article 11).			190 country members and 53 signatories (Basel Convention, 2011).
London Protocol (IMO, 2016)	Encourages scientific and technical cooperation within the organisation and with international bodies for minimising waste, improving production processes, disposing of, treating waste, etc. (Article 13). In particular to developing countries.	Promotes the acquisition of scientific advice. The Meeting of Contracting Bodies may invite appropriate advisory expert bodies (Article 18(3)), such as ACOPS and GESAMP.		Promotes regional and international cooperation (Articles 12,17).			53 states are parties to this protocol (40.31% World Tonnage) (IMO, 2022b)

MLG: multi-level governance; MARPOL: International Convention for the Prevention of Pollution from Ships; IMO: International Marine Organization; ACOPS: Advisory Committee on the Protection of the Sea; UNTC: United Nations Treaty Collection; SOCA: Subcommittee on Oceans and Coastal Areas; ICSPRO: Inter-Secretariat Committee on Scientific Programmes relating to Oceanography.

## Findings and discussion

### Findings

Comparing the international instruments revealed similarities and differences. The similarities lie in the emphasis on the prevention and control of marine pollution as one of the key objectives of these instruments. Yet there are differences in the types of sources and the plastic materials/items covered by the regulations. As shown in Table 2, instruments cover plastic ‘waste’ or ‘garbage’ that is discarded from ships (direct disposal, ship-borne operations) (LC/LP, MARPOL) as well as plastic wastes that are subject to transboundary movements (Basel Convention), but plastics are not specifically addressed in UNCLOS. Moreover, a common point is the difficulty of complying with the instruments (e.g. garbage amounts vary by ship category, legal ambiguity persists to define hazardous or non-hazardous plastic waste, etc.). Overall, these instruments play complementary roles in preventing and controlling marine pollution, but with fragmented authority.

It is evident from Table 3 that the revised documents have used SD features to connect the best expertise (*scientific cooperation and advice*) of international organisations and the advanced states in plastics pollution management with developing countries and inform the *foreign policy* – all instruments promote scientific research and technical collaboration to address the pollution of the marine environment. International organisations such as the Group of Experts on the Scientific Aspects of Marine Environmental Protection (GESAMP), using *scientific cooperation*, are best situated to provide *scientific advice* to *inform environmental policy*. The scientific communities have developed guidelines and methodologies for analysing marine macro- and micro-plastics, conducting research and providing relevant policy recommendations for member countries. Besides, support and utilisation of scientific research through international instruments are expected to indirectly advance science.

Protocols and conventions provide a platform for collaboration with the international community promoting regional and multilateral agreements which are expected to impact the *international relationships and foreign policy of state parties*. Moreover, establishing the international and regional instruments would not have been possible without pre-existing diplomatic links and agreements (*political capital*) between the participating countries, which in turn have *advanced scientific research*. The use of *political capital* is arguably not meeting all the needs of international scientific research cooperation, since the degree to which the different countries represent their own interests (*prioritise national needs*) is evidenced by the lack of commitment of country members to ratify the conventions, protocols and sign legally binding agreements.

### Discussion

In seeking to understand whether international governance from supra-national level has contributed to the progress of solving marine pollution, many have pointed out that strengthening international mechanisms is holding the key to progressing the solving of marine plastics pollution at international levels, focusing on the improvement of the obligatory character of international mechanisms (Vince and Hardesty, 2018), and development of a harmonious policy framework and coordinated policies (Dauvergne, 2018; Hugo, 2018; Pinto da Costa et al., 2020; Raubenheimer and McIlgorm, 2017). However, it remains unclear who fills the gaps in the instruments at a time when certain international cooperation, coordination and compliance mechanisms are absent due to fragmented authority and ambiguous coverage of plastic waste types and sources.

From an SD perspective, marine plastics pollution instruments have also been shaped by forms of governance that stretch across tiers supported by features of SD. This is not to deny the importance of strengthening international mechanisms. It is, however, to provide insights into the key factors encountered that prevent progress and that, under SD, could be redefined to take a more important role: the passive role of national authorities and representatives relying on the long-term development of a legal framework from supra-national and inter-regional levels rather than an active leadership. SD features such as scientific cooperation, advice and research supported by international relationship, foreign policy and political capital, have advanced international agreements. However, the power balance and political opportunity are crucial parts of the difficulties faced by international coordination (Ferraro and Failler, 2020). It often involves complex policies and goals, and only high-level stakeholders are involved – it can neither be assumed to exist nor solely relied upon (Rüffin, 2020). The *prioritisation of national needs* evidenced in the lack of commitment of country members to ratify the conventions, and difficulty in compliance and control of the sources of plastics pollution, act as a barrier for strengthening those international mechanisms. For example, LC adopted the control and prevention of marine pollution, but fewer parties than expected were able to contribute, and the growth of participants in ratifying the Convention was slower than anticipated (Harrison, 2017).

The opportunity here for leader nations is to revaluate their national needs in light of the potential benefits that could accrue from leading advancements in solving the problem of marine pollution including the enhancement of international relationships and national foreign policies – features embedded in SD. This leadership could benefit both country nations and marine plastics governance. However, cross-cutting leadership, supported by international agencies, needs to be also able to drive a multi-level regulatory framework for plastics (*political capital*) because the broad coverage of plastics-related issues under different levels of marine governance results in a lack of focus and priority with regard to plastics pollution and substantial hurdles in assessing the international and national instruments today. Initiatives from Japan (Dickella Gamaralalage and Onogawa, 2019) and Norway (IMO, 2019b) to assist developing countries in identifying opportunities to prevent and reduce marine litter and decrease the use of plastics in industries, which integrate various features of SD (e.g. scientific advice, scientific cooperation, political capital, etc.), could help to re-imagine the powers and capabilities of nations’ actors to develop leadership across multiple tiers.

In spite of all the efforts so far, the review in Table 2 showed that there is no single treaty instrument or policy framework to deal specifically with plastics marine litter within the current global governance. For instance, UNCLOS has many gaps regarding environmental protection (Bateman, 2007) and lacks specific standards for plastics pollution from both the sea and land-based sources. Besides, a multi-level regulation for plastics pollution is imperative, considering the growing consequences of marine debris pollution on humans and the environment (Ringbom and Henriksen, 2017), with a level of commitment equal to the global magnitude of the problem (Borrelle et al., 2017).

Moreover, this cross-cutting leadership supported by international agencies should also drive resources to increase treatment capabilities in the global south to absorb the growing waste generation. Although most of the international instruments promote scientific and technical cooperation, funding and other forms of partnership with the developed states are highly required (Sarma and Andersen, 2011); it is estimated that countries in Southeast Asia could only manage the disposal and burning of 25%–75% of the imported plastics waste (Law et al., 2020). International bureaucracies may have a wider role in international climate negotiations by interacting with sub-national and non-state actors (Hickmann et al., 2021). The critical issues of waste management are faced not only by the developing world but also by the most industrialised nations that lack waste management treatment capabilities (D’Amato et al., 2019). However, in the long term, the sole improvement of treatment capabilities of waste management does not appear to provide a solution by itself; the recycling system only perpetuates the problem, driving more plastics production.

Focusing solely on the recycling system leads to a discussion about the discursive approach framing plastics in the international agreements as ‘waste’ or ‘garbage’ – fostering economic growth. The need to achieve higher economic growth (Everett et al., 2010) has shaped the debate about marine plastics, in which policies related to environmental management are often sidelined. Placing the plastics pollution issue as a national priority is challenged by mainly economic considerations (Barrowclough and Deere Birkbeck, 2020), ‘trapping’ countries in the middle of a trade-off between attaining economic development and preserving the environment. Especially as ‘extensive structural change in the economic system is required to achieve sustainable use of natural resources; it is not possible to “develop to sustainability”’ (Cumming and Von Cramon-Taubadel, 2018: 9533). For instance, this interpretation of the plastics marine problem based on economic growth has shaped the continued waste exchange from the global north to the south, the (lack of) recycling systems, and the prioritisation of corporate self-regulation. In this context, although marine plastics pollution continues to be framed under economic discourses, it will perpetuate the problem.

### Theoretical implications

This paper contributes novel insights into SD literature by specifying the main salient features that can be used either individually or collectively to characterise ‘science’ and ‘diplomacy’ functions. In this context, the SD features can shed light on the opportunities and limitations facing transboundary environmental problems, particularly identifying forms of governance that are supported by SD features that can potentially emerge to be more relevant. As a newly emerging area, however, SD does not have clearly delineated legal jurisdictions. Recent attempts from Rüffin (2020), based on the theoretical notions of MLG from Marks and Hooghe (Hooghe and Marks, 2003; Marks and Hooghe, 2004), conceptualise SD as a Type II MLG system in which ‘Policies concerning SD might touch upon innumerable jurisdictions’ (Rüffin, 2020: 3).

In MLG Type I, a clear hierarchy exists between distinct tiers of governance. In theory, it seems feasible to link the established hierarchy from global spheres to individual nations concerning plastics pollution. Thus, all nation-states have the powers and capabilities to pursue an agenda for reducing and managing plastics waste within the national remit. However, in practice, the technical knowledge and financial capabilities gap, and political and economic interests within states, limit the ‘willingness’ of nations to implement the international agreements into concrete policies within their respective countries. For example, the waste management policy concerning plastics depends on funding availability. In part, this forces us to first reflect on the potential changes of responsibilities and capabilities of emergent actors of cross-level governance, leading to paying more attention to MLG Type II – which is more fluid, drawing attention to the importance of analyses of the changing nations’ collaboration level with different ‘spheres’ of authorities.

### Implications for policymaking

The cost of reducing plastics pollution and the current international mechanisms generate no incentives for individual countries to tackle this urgent problem. In this context, incorporating an SD features approach may help to enhance foreign policies and national strategies, international cooperation and scientific efforts. Particularly, we argue that cross-cutting leading efforts to engage more countries in different parts of the world, specifically with developing countries, with less adequate scientific and technological capabilities to solve plastics pollution, could benefit the leaders’ foreign policy. For example, in this context, following SD-MLG could help reshape the UK's foreign policy into what has recently been envisioned as a scientific leader, thus expanding its engagement with international partners, and offering more diplomatic manoeuvres after leaving the European Union.

### Limitations and future research

In advancing environmental governance, the case of plastics pollution in the ocean based on a documentary review and analysis draws attention to the link between SD salient features and international policies for marine plastics pollution. Although this methodology has its merits to accomplish the review and the analysis, it may be biased in representing a complete account of the problem. Further studies with empirical data from interviews with the main international stakeholders should investigate the subsequent question of whether the SD perspective and its application in Type II MLG are feasible, and its implications, taking into account the countries dependency on plastic production and consumption. At the same time, it would be worthwhile reviewing its implications for alternative plastic materials such as bioplastics and their supply chains issues described by Beltran et al. (2021) and Liliani et al. (2020), and comparing similar studies for transboundary problems. For instance, the battles between science and policy played out in relation to the COVID-19 pandemic and the climate change agenda.

## Conclusion

This paper contributes by identifying key salient features that can be used individually or collectively to characterise ‘science’ and ‘diplomacy’ functions, providing new insights into SD literature and presenting a framework that can also be applied to transboundary issues. This paper also presents a documentary analysis that integrates the SD framework into understanding international instruments’ role in reducing and mitigating marine plastic pollution – a topic which has received little research attention. It has been noted that there is a research gap between what different international instruments under the term SD mean, and whether SD is truly effective in addressing marine plastic pollution.

The documentary analysis revealed similarities and differences between international instruments and their SD features. The study found that although there is a strong emphasis on the prevention and control of marine pollution in the international agreements and they are complementary; a common difficulty is establishing compliance and control mechanisms. More importantly, it is unclear who is responsible for filling the gaps in instruments due to fragmented authority and various coverage of plastic waste types and sources. The documentary analyses also added a new SD feature, the degree to which different countries represent their own interests in terms of ‘prioritising national needs’. This is a challenge to strengthen international mechanisms, increase commitment to ratify and comply with the conventions, and controlling plastic pollution sources. In addition, it recognises dominant approaches that have framed plastics as waste rather than a resource.

Although it acknowledges this challenge, the paper argues that it can also be viewed as an opportunity for leader nations to reassess their national priorities in light of the potential benefits that could accrue from advancing marine pollution solutions, including enhanced international relations and national foreign policies. In this context, SD needs changes of responsibilities and capabilities of emergent actors of cross-level governance, leading to more attention being paid to MLG Type II. However, empirical research is needed to further explore the effects of this leadership and its effectiveness in addressing marine pollution, taking into account the countries' path dependency on plastic production and consumption.

## Supplemental Material

sj-xlsx-1-ras-10.1177_00208523231183909 - Supplemental material for Rethinking marine plastics pollution: Science diplomacy and multi-level governance Supplemental material, sj-xlsx-1-ras-10.1177_00208523231183909 for Rethinking marine plastics pollution: Science diplomacy and multi-level governance by Macarena Beltran, Benny Tjahjono, Thomas Noto Suoneto, Rakyan Tanjung and Jorge Julião in International Review of Administrative Sciences

## References

[bibr1-00208523231183909] Agamuthu P Mehran S Norkhairah A , et al. (2019) Marine debris: A review of impacts and global initiatives. Waste Management & Research 37(10): 987–1002. 31084415 10.1177/0734242X19845041

[bibr2-00208523231183909] Ahmed JU (2010) Documentary research method: New dimensions. Indus Journal of Management and Social Sciences 4(1): 1–14.

[bibr3-00208523231183909] Atkinson P Coffey A (2010) Analysing documentary realities. In: Silverman D (ed.) Qualitative Research: Theory, Method and Practice. London: SAGE.

[bibr4-00208523231183909] Barrowclough D Deere Birkbeck C (2020) Transforming the global plastics economy: The political economy and governance of plastics production and pollution (no. 142). Global Economic Governance Programme (GEG) Working Paper, University of Oxford.

[bibr5-00208523231183909] Basel Convention (2011) Parties to the Basel Convention on the control of transboundary movements of hazardous wastes and their disposal. Available at: http://www.basel.int/?tabid=4499#US17 (accessed 21 June 2023).

[bibr6-00208523231183909] Basel Convention (2019a) Basel Convention plastic waste amendments. Available at: http://www.basel.int/Implementation/Plasticwaste/Amendments/Overview/tabid/8426/Default.aspx (accessed 21 June 2023).

[bibr7-00208523231183909] Basel Convention (2019b) Questions and answers related to the Basel Convention plastic waste amendments. Available at: http://www.basel.int/Implementation/Plasticwaste/PlasticWasteAmendments/FAQs/tabid/8427/Default.aspx (accessed 21 June 2023).

[bibr800-00208523231183909] Bateman S . (2007) UNCLOS and its limitations as the foundation for a regional maritime security regime. The Korean Journal of Defense Analysis 19(3): 27–56.

[bibr8-00208523231183909] Beltran M Tjahjono B Bogush A , et al. (2021) Food plastic packaging transition towards circular bioeconomy: A systematic review of literature. Sustainability 13(7): 1–24.

[bibr9-00208523231183909] Boekholt P Edler J Cunningham P , et al. (2009) Drivers of International Collaboration in Research. Luxembourg: Publications Office of the European Commission.

[bibr10-00208523231183909] Borrelle SB Rochman CM Liboiron M , et al. (2017) Why we need an international agreement on marine plastic pollution. Proceedings of the National Academy of Sciences of the United States of America 114: 9994–9997. 28928233 10.1073/pnas.1714450114 PMC5617320

[bibr11-00208523231183909] Chen C-L (2015) Regulation and management of marine litter. In: Bergmann M Gutow L Klages M (eds) Marine Anthropogenic Litter. Cham: Springer, 395–428.

[bibr12-00208523231183909] Copeland D (2015) Bridging the chasm. Science & Diplomacy 4(3): 1–6.

[bibr13-00208523231183909] Cumming GS von Cramon-Taubadel S (2018) Linking economic growth pathways and environmental sustainability by understanding development as alternate social-ecological regimes. Proceedings of the National Academy of Sciences of the United States of America 115: 9533–9538. 30185564 10.1073/pnas.1807026115 PMC6156676

[bibr14-00208523231183909] D’Amato A Paleari S Pohjakallio M , et al. (2019) Plastics Waste Trade and the Environment. Boeretang: European Topic Centre Waste and Materials in a Green Economy.

[bibr15-00208523231183909] Dauvergne P (2018) Why is the global governance of plastic failing the oceans? Global Environmental Change 51: 22–31.

[bibr16-00208523231183909] Dickella Gamaralalage PJ Onogawa K (2019) Strategies to Reduce Marine Plastics Pollution from Land-based Sources in Low and Middle-income Countries. Nairobi: United Nations Environment Programme.

[bibr17-00208523231183909] Everett T Ishwaran M Ansaloni GP (2010) *Economic growth and the environment*. MPRA Paper No. 23585. London: DEFRA.

[bibr18-00208523231183909] Fähnrich B (2017) Science diplomacy: Investigating the perspective of scholars on politics–science collaboration in international affairs. Public Understanding of Science 26(6): 688–703. 26721551 10.1177/0963662515616552

[bibr19-00208523231183909] Ferraro G Failler P (2020) Governing plastic pollution in the oceans: Institutional challenges and areas for action. Environmental Science Policy 112: 453–460.

[bibr20-00208523231183909] Gluckman PD Turekian VC Grimes RW , et al. (2017) Science diplomacy: A pragmatic perspective from the inside. Science & Diplomacy 6(4): 1–13.

[bibr21-00208523231183909] Gold M Mika K Horowitz C , et al. (2014) Stemming the tide of plastic litter: A global action agenda. Tulane Environmental Law Journal 27: 165–203.

[bibr22-00208523231183909] Harden-Davies H (2018) The next wave of science diplomacy: Marine biodiversity beyond national jurisdiction. ICES Journal of Marine Science 75(1): 426–434.

[bibr23-00208523231183909] Harrison J (2017) Ocean dumping. Yearbook of International Environmental Law 26: 196–204.

[bibr24-00208523231183909] Hickmann T Widerberg O Lederer M , et al. (2021) The United Nations Framework Convention on Climate Change Secretariat as an orchestrator in global climate policymaking. International Review of Administrative Sciences 87(1): 21–38.

[bibr25-00208523231183909] Hooghe L Marks G (2003) Unraveling the central state, but how? Types of multi-level governance. American Political Science Review 97(2): 233–243.

[bibr26-00208523231183909] Hugo TG (2018) The case for a treaty on marine plastic pollution. Available at: http://intlaw.no/wp-content/uploads/2018/11/The-case-for-a-TMPP-Nov-2018-WEB.pdf (accessed 16 March 2022).

[bibr27-00208523231183909] International Maritime Organization (2013) MARPOL – How to Do It. London: IMO.

[bibr28-00208523231183909] International Maritime Organization (2016) Convention on the prevention of marine pollution by dumping of wastes and other matter, 1972. London Convention, 1972, with Protocol 1996. London: IMO.

[bibr29-00208523231183909] International Maritime Organization (2019a) *Brief history of IMO*. Available at: https://www.imo.org/en/About/HistoryOfIMO/Pages/Default.aspx (accessed 21 June 2023).

[bibr30-00208523231183909] International Maritime Organization (2019b) Global project launched to tackle plastics litter from ships and fisheries. Available at: https://www.imo.org/en/MediaCentre/PressBriefings/Pages/32-GloLitter-signing.aspx (accessed 21 June 2023).

[bibr31-00208523231183909] International Maritime Organization (2022a) MARPOL Consolidated Edition 2022. IMO. https://vp.imo.org/index.html (accessed 9 February 2023

[bibr32-00208523231183909] International Maritime Organization (2022b) Status of Convention ratifications by state (flag) and ratifications by treaty as of 2022-10-21. London: IMO.

[bibr33-00208523231183909] Kleinhaus K Al-Sawalmih A Barshis DJ , et al. (2020) Science, diplomacy, and the Red Sea’s unique coral reef: It’s time for action. Frontiers in Marine Science 7.

[bibr34-00208523231183909] Law KL Starr N Siegler TR , et al. (2020) The United States’ contribution of plastic waste to land and ocean. Science Advances 6(44): eabd0288. 10.1126/sciadv.abd0288 PMC7608798 33127684

[bibr35-00208523231183909] LilianiTjahjono B Cao D (2020) Advancing bioplastic packaging products through co-innovation: A conceptual framework for supplier–customer collaboration. Journal of Cleaner Production 252: 119861.

[bibr36-00208523231183909] Lord KM Turekian VC (2007) Time for a new era of science diplomacy. Science 315(5813): 769–770. 17289962 10.1126/science.1139880

[bibr37-00208523231183909] Marks G Hooghe L (2004) Contrasting visions of multi-level governance. In: Bache I Flinders M (eds) Multi-Level Governance. Oxford: Oxford University Press, 15–30.

[bibr38-00208523231183909] Moedas C (2016) Science diplomacy in the European Union. Science & Diplomacy 5(1): 1–9.

[bibr39-00208523231183909] Paglia E (2021) The Swedish initiative and the 1972 Stockholm conference: The decisive role of science diplomacy in the emergence of global environmental governance. Humanities and Social Sciences Communications 8(2): 1–10.

[bibr40-00208523231183909] Pan M Huntington HP (2016) A precautionary approach to fisheries in the central Arctic Ocean: Policy, science, and China. Marine Policy 63: 153–157.

[bibr41-00208523231183909] Patman RG Davis LS (2017) Science diplomacy in the Indo-Pacific region: A mixed but promising experience. Politics Policy 45(5): 862–878.

[bibr42-00208523231183909] Pinto da Costa J Rocha-Santos T Duarte A (2020) The Environmental Impacts of Plastics and Micro-Plastics use, Waste, and Pollution: EU and National Measures. Brussels: Policy Department for Citizens’ Rights and Constitutional Affairs. European Parliament.

[bibr43-00208523231183909] Polejack A (2021) The importance of ocean science diplomacy for ocean affairs, global sustainability, and the UN decade of ocean science. Frontiers in Marine Science 8: 664066.

[bibr44-00208523231183909] Polejack A Gruber S Wisz MS (2021) Atlantic Ocean science diplomacy in action: The pole-to-pole All Atlantic Ocean Research Alliance. Humanities and Social Sciences Communications 8: article 52.

[bibr45-00208523231183909] Raubenheimer K McIlgorm A (2017) Is the Montreal protocol a model that can help solve the global marine plastic debris problem? Marine Policy 81: 322–329.

[bibr46-00208523231183909] Rayner J Howlett M (2009) Conclusion: Governance arrangements and policy capacity for policy integration. Policy and Society 28(2): 165–172.

[bibr47-00208523231183909] Ringbom H Henriksen T (2017) Governance Challenges, Gaps and Management Opportunities in Areas Beyond National Jurisdiction. Washington, DC: Global Environment Facility-Scientific and Technical Advisory Panel.

[bibr48-00208523231183909] Royal Society (2010) New frontiers in science diplomacy: Navigating the changing balance of power. RS policy document 01/10. London: Science Policy Centre London.

[bibr49-00208523231183909] Rüffin N (2020) EU science diplomacy in a contested space of multi-level governance: Ambitions, constraints and options for action. Research Policy 49(1): 103842.

[bibr50-00208523231183909] Ruffini P-B (2020) Conceptualizing science diplomacy in the practitioner-driven literature: A critical review. Humanities and Social Sciences Communications 7: article 124.

[bibr51-00208523231183909] Sarma KM Andersen S (2011) Science and diplomacy: Montreal protocol on substances that deplete the ozone layer. In: Berkman PA Lang MA Walton DWH (eds) Science Diplomacy: Antarctica, Science, the Governance of International Spaces. Washington, DC: Smithsonian Institution Scholarly Press, 123–132.

[bibr52-00208523231183909] Shaw S Elston J Abbott S (2004) Comparative analysis of health policy implementation. Policy Studies 25(4): 259–266.

[bibr53-00208523231183909] Teff-Seker Y Mackelworth PC Vega Fernández T , et al. (2020) Do alternative dispute resolution (ADR) and track two processes support transboundary marine conservation? Lessons from six case studies of maritime disputes. Frontiers in Marine Science 7: 593265.

[bibr54-00208523231183909] Tight M (2019) Documentary Research in the Disciplines. Documentary Research in the Social Sciences. London: SAGE Publications.

[bibr55-00208523231183909] Turekian V (2018) The evolution of science diplomacy. Global Policy 9(3): 5–7.32863889

[bibr56-00208523231183909] United Nations (2001) Oceans and the law of the sea: Report of the Secretary-General. New York: United Nations.

[bibr57-00208523231183909] United Nations Convention on the Law of the Sea (1982) United Nations Convention on the Law of the Sea of 10 December 1982. https://vp.imo.org/index.html. (accessed 9 February 2023).

[bibr58-00208523231183909] United Nations Environment Programme (2014) Basel Convention on the Control of Transboundary Movements of Hazardous Wastes and their disposal. Geneva: United Nations Environment Programme.

[bibr59-00208523231183909] United Nations Environment Programme (2023) About UN Environment Programme. Available at: https://www.unep.org/about-un-environment (accessed 21 June 2023).

[bibr60-00208523231183909] United Nations Treaty Collection (2023) Status of treaties. Chapter XXI. Law of the sea. Available at: https://treaties.un.org/pages/ViewDetailsIII.aspx?src=TREATY&mtdsg_no=XXI-6&chapter=21&Temp=mtdsg3&clang=_en (accessed 21 June 2023).

[bibr61-00208523231183909] Vince J Hardesty BD (2018) Governance solutions to the tragedy of the commons that marine plastics have become. Frontiers in Marine Science 5: 214.

[bibr62-00208523231183909] Weiss C (2015) How do science and technology affect international affairs? Minerva 53: 411–430. 10.1007/s11024-012-9191-9 PMC3283766 22389530

